# EOMES is essential for antitumor activity of CD8^+^ T cells in chronic lymphocytic leukemia

**DOI:** 10.1038/s41375-021-01198-1

**Published:** 2021-03-17

**Authors:** Laura Llaó-Cid, Philipp M. Roessner, Vicente Chapaprieta, Selcen Öztürk, Tobias Roider, Marie Bordas, Ana Izcue, Dolors Colomer, Sascha Dietrich, Stephan Stilgenbauer, Bola Hanna, José Ignacio Martín-Subero, Martina Seiffert

**Affiliations:** 1 Molecular Genetics, German Cancer Research Center (DKFZ), Heidelberg, Germany; 2 Faculty of Biosciences, University of Heidelberg, Heidelberg, Germany; 3 Institut d’Investigacions Biomèdiques August Pi I Sunyer (IDIBAPS), Barcelona, Spain; 4 Department of Medicine V, Hematology, Oncology and Rheumatology, University of Heidelberg, Heidelberg, Germany; 5 Max-Planck-Institute of Immunobiology and Epigenetics, Freiburg, Germany; 6 Center for Chronic Immunodeficiency, University Medical Center Freiburg and University of Freiburg, Freiburg, Germany; 7 Hematopathology Section, Hospital Clinic, Barcelona, Spain; 8 Departament de Fonaments Clínics, Universitat de Barcelona, Barcelona, Spain; 9 Centro de Investigación Biomédica en Red de Cáncer (CIBERONC), Barcelona, Spain; 10 Department of Internal Medicine III, University of Ulm, Ulm, Germany; 11 Department of Internal Medicine I, Saarland University, Homburg, Germany; 12 Institució Catalana de Recerca i Estudis Avançats (ICREA), Barcelona, Spain; 13 Present Address: Institute of Molecular Medicine, University Hospital RWTH Aachen, Aachen, Germany

**Keywords:** Chronic lymphocytic leukaemia, Immunosurveillance, Preclinical research

## Abstract

Genome-wide association studies identified a single-nucleotide polymorphism (SNP) affecting the transcription factor Eomesodermin (EOMES) associated with a significantly increased risk to develop chronic lymphocytic leukemia (CLL). Epigenetic analyses, RNA sequencing, and flow cytometry revealed that EOMES is not expressed in CLL cells, but in CD8^+^ T cells for which EOMES is a known master regulator. We thus hypothesized that the increased CLL risk associated with the *EOMES* SNP might be explained by its negative impact on CD8^+^ T-cell-mediated immune control of CLL. Flow cytometry analyses revealed a higher EOMES expression in CD8^+^ T cells of CLL patients compared to healthy individuals, and an accumulation of PD-1^+^ EOMES^+^ CD8^+^ T cells in lymph nodes rather than blood or bone marrow in CLL. This was in line with an observed expansion of EOMES^+^ CD8^+^ T cells in the spleen of leukemic Eµ-TCL1 mice. As EOMES expression was highest in CD8^+^ T cells that express inhibitory receptors, an involvement of EOMES in T-cell exhaustion and dysfunction seems likely. Interestingly, *Eomes*-deficiency in CD8^+^ T cells resulted in their impaired expansion associated with decreased CLL control in mice. Overall, these observations suggest that EOMES is essential for CD8^+^ T-cell expansion and/or maintenance, and therefore involved in adaptive immune control of CLL.

## Introduction

Genome-wide association studies (GWAS) have shown that a single-nucleotide polymorphism (SNP) affecting the transcription factor Eomesodermin (*EOMES*) is associated with a significantly increased risk to develop chronic lymphocytic leukemia (CLL) [1]. Epigenetic analyses have revealed, however, that the SNP associated with *EOMES* is located in a heterochromatic region in CLL cells and normal B cells, independently of the presence or absence of the risk variant [2]. This finding contrast other risk loci predisposing to CLL, which are frequently located in regulatory elements in CLL cells [2], and suggests that *EOMES* may be related to CLL development through its expression in microenvironmental cells rather than in the neoplastic clone itself.

EOMES is a transcription factor that belongs to the conserved T-box gene family and regulates T-cell development and function [3–5]. As a master regulator of CD8^+^ effector and memory T cells, EOMES is critical for T-cell-mediated immune responses against pathogens [4, 6–8]. An accumulation of EOMES-expressing CD8^+^ T cells that show characteristics of exhausted cells was observed in several tumor entities [9–12]. T-cell exhaustion was first described in chronic viral infections as a dysfunctional state of CD8^+^ T cells that develops due to persistent antigen exposure and is characterized by the co-expression of multiple inhibitory receptors, including programmed cell death 1 (PD-1), lymphocyte activating gene 3 (LAG3), T-cell immunoreceptor with Ig and ITIM domains (TIGIT), and cluster of differentiation 244 (CD244), defective effector function, and an altered transcriptional and epigenetic state [9, 13–16]. Based on this, a role for EOMES in T-cell dysfunction following continuous activation of CD8^+^ T cells was suggested [15, 17].

CLL development is not only associated with alterations of the neoplastic cells but also with alterations of the T-cell compartment, including an enrichment of effector (T_EF_) and effector memory (T_EM_) T cells and leukemia-induced defects in immune synapse formation [18–21]. Oligoclonally expanded CD8^+^ T cells have been shown to control leukemia development in the Eµ-TCL1 adoptive transfer (AT) mouse model for CLL [20], but also to acquire a dysfunctional or exhausted state [18, 22]. We hypothesized that EOMES is involved in T-cell dysfunction in CLL, and the increased risk for CLL development in individuals with a SNP in the *EOMES* locus is due to a reduced ability of CD8^+^ T cells to control leukemia progression.

Here, we describe the accumulation of EOMES^+^ CD8^+^ T cells in lymph nodes (LNs) and blood of CLL patients as well as in the Eµ-TCL1 mouse model. Importantly, by co-transferring CD8^+^ T cells and Eµ-TCL1 leukemia cells in immunodeficient mice, we provide evidence for an EOMES-dependent T-cell control of leukemia progression.

## Results

### EOMES expression is increased in CD8^+^ T cells in CLL patients

**Fig. 1:**
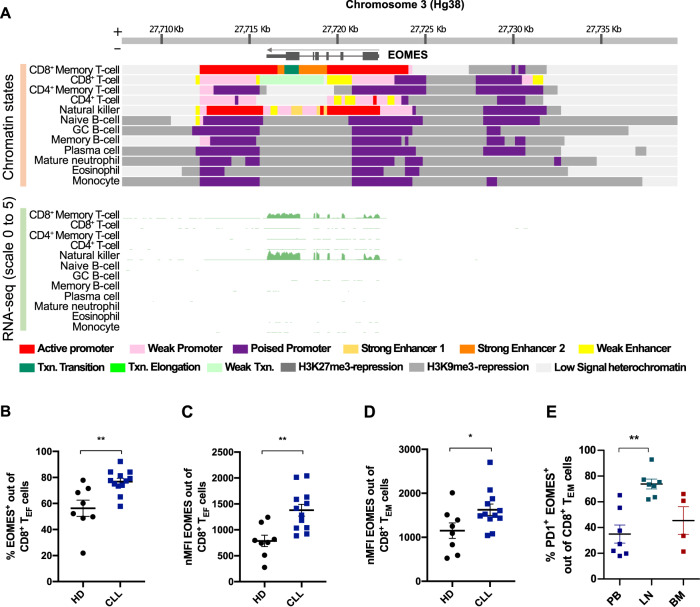
EOMES expression is increased in CD8^+^ T cells from CLL patients. **A** *EOMES* epigenomic landscape in hematopoietic cells. Chromatin state at the *EOMES* locus in hematopoietic cells of healthy individuals, including B cells at diverse differentiation stages. Histone marks from the Blueprint project were downloaded and chromatin states were determined. **B**–**D** EOMES expression was analyzed by flow cytometry in CD8^+^ T cells from peripheral blood of CLL patients (*n* = 12) and healthy donors (HD, *n* = 8). **B** Percentage of EOMES^+^ cells out of T_EF_ CD8^+^ T cells from PB of CLL patients and healthy donors. Normalized mean fluorescence intensity (nMFI) of EOMES in **C** CD8^+^ T_EF_ cells and **D** CD8^+^ T_EM_ cells from CLL and HD. **E** Percentage of EOMES^+^ PD-1^+^ cells out of CD8^+^ T_EM_ cells in paired peripheral blood (PB, *n* = 7), lymph node (LN, *n* = 7), and bone marrow (BM, *n* = 4) samples of CLL patients. Gating strategy for CLL cells and T-cell subsets is provided in the Supplementary information. Mean ± SEM is shown in all graphs, with each dot representing one patient or donor. One-way ANOVA was used to investigate the differences in % EOMES^+^ PD-1^+^ cells between PB, LN, and BM samples. Mann–Whitney test was used for statistical analysis. *p value < 0.05, **p value < 0.01.

In order to identify the cell types that have a euchromatic state at the *EOMES* locus, we analyzed the chromatin status and RNA expression of the *EOMES* locus in hematopoietic cells, including B cells and T cells at diverse differentiation states. More specifically, the chromatin states derived from the integration of six marks (i.e., H3K4me3, H3K4me1, H3K27ac, H3K36me3, H3K27me3, and H3K9me3) together with RNA-sequencing data from the reference epigenomes generated in the Blueprint project database (http://www.blueprint-epigenome.eu) were investigated. The *EOMES* epigenomic and transcriptomic profiling indicated that active chromatin at this locus is detectable solely in T cells and NK cells, with CD8^+^ memory T cells being particularly active. In contrast, B cells at different maturation stages presented a heterochromatic configuration and no RNA expression of *EOMES* (Fig. 1A). Given that CD8^+^ T cells accumulate in peripheral blood (PB) of patients, and play a significant role in CLL development [18, 23–25], we next investigated protein expression of EOMES in malignant B cells as well as CD8^+^ T cells from PB of CLL patients. Using flow cytometric analyses, we detected no EOMES expression in CLL cells, whereas the average percentage of EOMES-positive CD8^+^ T cells in PB from CLL patients was 61.5 ± 8.39% (Supplementary Fig. 1A–C).

To assess potential abnormalities in EOMES^+^ T cells in CLL, PB samples of CLL patients and age-matched healthy donors (HD) were analyzed. The frequency of EOMES^+^ cells in CD8^+^ T_EM_, central memory (T_CM_) and naive (T_N_) T-cell subsets was similar in CLL and HD (Supplementary Fig. 1D), but a significantly higher percentage of EOMES^+^ cells within the CD8^+^ T_EF_ subset was observed in CLL patients (Fig. 1B). We further examined T_EM_ and T_EF_ subsets, which exhibited higher frequencies of EOMES^+^ cells compared to T_CM_ and T_N_ subsets. Interestingly, higher expression levels of EOMES, as determined by normalized mean fluorescence intensity analysis in flow cytometry, were measured in T_EF_ and T_EM_ CD8^+^ T cells from CLL versus HD blood samples (Fig. 1C, D). Within the CD8^+^ T_EM_ population, EOMES expression levels were higher in PD-1^+^ versus PD-1^−^ cells of both CLL and HD (Supplementary Fig. 1E), confirming the relevance of EOMES in activated T cells and suggesting that, similarly as in other tumor entities [6, 26], EOMES accumulates in exhausted T cells in CLL. Yet, EOMES^+^ PD-1^+^ CD8^+^ T cells, which are potentially exhausted cells, were not more frequent in the T_EM_ population in PB of CLL patients compared to HD (Supplementary Fig. 1F). Because chronic T-cell activation and exhaustion in CLL has been shown to mainly occur in secondary lymphoid organs where T cells and CLL cells interact [20, 27], we measured the frequencies of these cells in paired LN, PB, and bone marrow (BM) samples of CLL patients. Interestingly, we observed that EOMES^+^ PD-1^+^ cells constitute the major CD8^+^ T_EM_ subset in CLL LNs and are present in significantly higher frequencies in LNs compared to PB of the patients (Fig. 1E). The percentage of this cell type was, however, similar in PB and BM samples (Fig. 1E). The inclusion of reactive LN samples from individuals without cancer revealed comparable frequencies of EOMES^+^ PD-1^+^ T_EM_ in this tissue as in CLL LNs (Supplementary Fig. 1G). In contrast, EOMES expression levels were higher in T_EF_ CD8^+^ T cells in CLL LN (Supplementary Fig. 1H).

Altogether, these results demonstrate enhanced expression of EOMES in CD8^+^ T cells of CLL patients compared to healthy individuals. In addition, we show that PD-1^+^ T cells express high levels of EOMES and that these potentially exhausted PD-1^+^ EOMES^+^ T cells accumulate in LNs rather than PB. As gene and protein expression of *EOMES* are not detectable in CLL cells, EOMES-expressing T cells in the tumor microenvironment (TME) could underline the association between the *EOMES*-SNPs and CLL development.

### EOMES^+^ CD8^+^ T cells accumulate in the Eµ-TCL1 mouse model of CLL where EOMES is linked to CD8^+^ T-cell exhaustion

**Fig. 2:**
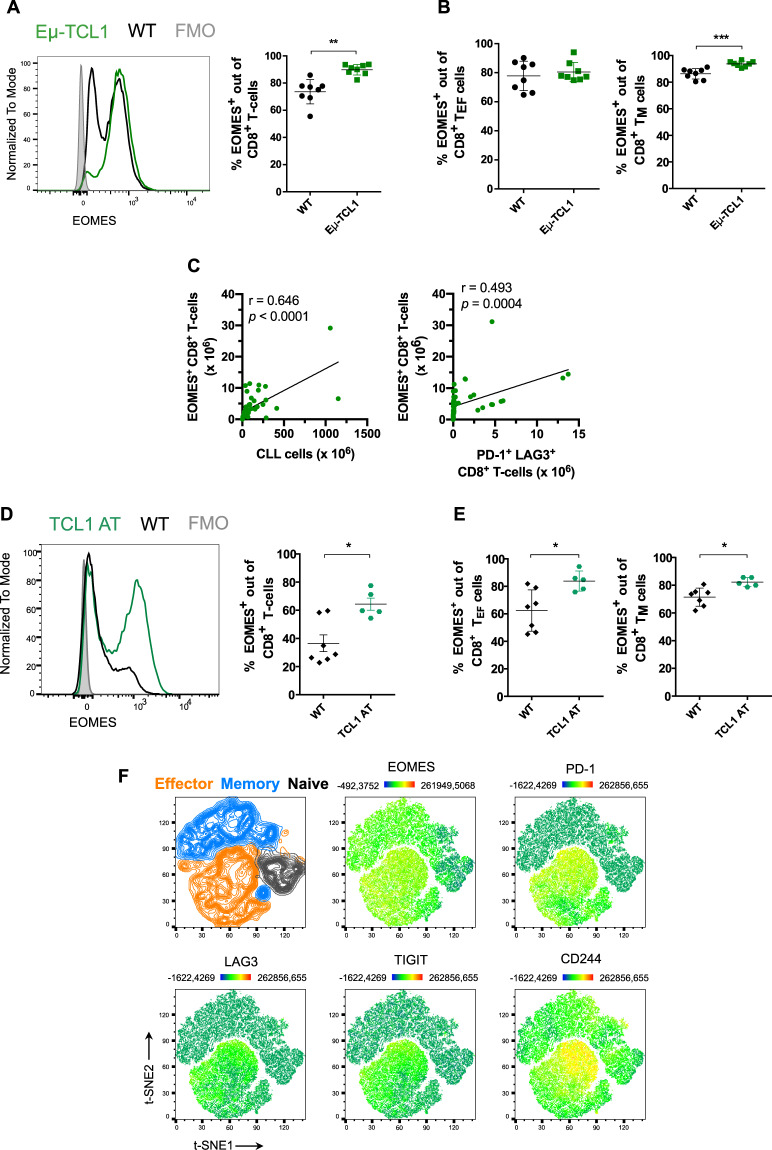
EOMES^+^ CD8^+^ T cells showing features of exhaustion accumulate in the Eµ-TCL1 mouse model of CLL. **A** Splenocytes of primary heterozygous Eµ-TCL1 leukemic mice (*n* = 8) and wild-type (WT, *n* = 8) littermates at a median age of 65 weeks were analyzed by flow cytometry. Representative histogram and percentage of EOMES^+^ cells out of total CD8^+^ T cells are depicted. **B** Percentage of EOMES^+^ cells among CD8^+^ T_EF_ cells (left) and CD8^+^ T_M_ cells (right). **C** Pearson correlation of absolute counts of EOMES^+^ CD8^+^ T cells and CLL cells (left), as well as PD-1^+^ LAG3^+^ CD8^+^ T cells (right) in spleen of Eµ-TCL1 leukemic mice (*n* = 48). **D** EOMES expression in splenic CD8^+^ T cells of mice after adoptive transfer of TCL1 leukemia (TCL1 AT, *n* = 5) with end-stage disease and WT littermates (*n* = 7) was analyzed by flow cytometry. Representative histogram and percentage of EOMES^+^ cells out of total CD8^+^ T cells are shown. **E** Percentage of EOMES^+^ cells out of CD8^+^ T_EF_ cells (left) and of CD8^+^ T_M_ cells (right). **F** Splenic CD8^+^ T cells from TCL1 AT mice (*n* = 4) were concatenated and clustered using t-SNE algorithm based on the expression of CD127, CD44, EOMES, PD-1, LAG3, TIGIT, and CD244. Effector, memory, and naïve subsets were defined based on CD127 and CD44 expression (upper left panel, see “Materials and methods”). EOMES, PD-1, LAG3, TIGT, and CD244 protein levels in clustered CD8^+^ T cells are shown (middle and right upper panels, and lower panels). Graphs show mean with SEM, with each dot representing one mouse. Statistical analysis was performed using the Mann–Whitney’s test. *p value < 0.05, **p value < 0.01, ***p value < 0.001.

Next, we investigated whether the Eµ-TCL1 mouse model of CLL is a good tool to examine the role of EOMES-expressing CD8^+^ T cells. Interestingly, a higher proportion of total CD8^+^ T cells expressing EOMES was observed in Eµ-TCL1 mice compared to age- and sex-matched wild-type (WT) mice, as analyzed by flow cytometry (Fig. 2A and Supplementary Fig. 2A). This difference might be due to the previously described skewing of T cells toward antigen-experienced memory T cells (T_M_) in CLL developed by Eµ-TCL1 mice (Supplementary Fig. 2B). In line with this, T_M_ cells, but not T_EF_, expressed higher levels of EOMES in Eµ-TCL1 mice compared to WT controls (Fig. 2B). To investigate a potential association of EOMES expression with T-cell exhaustion and tumor burden, we correlated the numbers of EOMES^+^ CD8^+^ T cells with the numbers of exhausted T cells as well as CLL cells in a cohort of leukemic Eµ-TCL1 mice displaying a wide range of tumor load from 10 to 95%. This showed that total CD8^+^ T cells as well as EOMES^+^ CD8^+^ T cells positively correlated with disease load (Supplementary Fig. 2C and Fig. 2C), and that a positive correlation of EOMES-expressing CD8^+^ T cells with cells co-expressing the inhibitory receptors PD-1 and LAG3, which are markers of T-cell exhaustion, exists (Fig. 2C). Furthermore, the CD8^+^ T-cell subset with PD-1 and LAG3 co-expression showed the highest expression of EOMES compared to effector, memory and naïve T-cell subsets, as well as compared to double negative effector and memory CD8^+^ T cells (Supplementary Fig. 2D, E).

We further examined whether EOMES^+^ CD8^+^ T cells accumulate after AT of Eµ-TCL1 leukemic cells into syngeneic WT animals (TCL1 AT). Similarly to the Eµ-TCL1 transgenic mice, a decrease of naïve T cells along with an increase of memory T cells occurred in these mice upon tumor development, in addition to higher percentages of effector T cells compared to WT mice (Supplementary Fig. 2F). Of note, increased levels of EOMES-expressing T cells in both effector and memory subsets of TCL1 AT mice, in contrast to non-leukemic control mice were detected (Fig. 2D, E), which confirms previously published data [20, 28]. We subsequently assessed EOMES expression levels together with the exhaustion markers PD-1, LAG3, TGIT, and CD244. Clustering of CD8^+^ T cells using t-SNE (t-distributed stochastic neighbor embedding) algorithm revealed a subset of effector T cells co-expressing all of these marker proteins, denoting them as exhausted T cells (Fig. 2F). Remarkably, EOMES expression was highest in these identified exhausted T cells (Fig. 2F and Supplementary Fig. 2G, H).

Overall, the Eµ-TCL1 and TCL1 AT mouse models of CLL recapitulate the accumulation of EOMES^+^ CD8^+^ T cells in the TME of CLL patients. In addition, EOMES expression is highest in CD8^+^ T cells that express inhibitory receptors, suggesting an involvement of EOMES in T-cell exhaustion.

### Lack of EOMES in CD8^+^ T cells leads to their impaired expansion and decreased tumor control

**Fig. 3:**
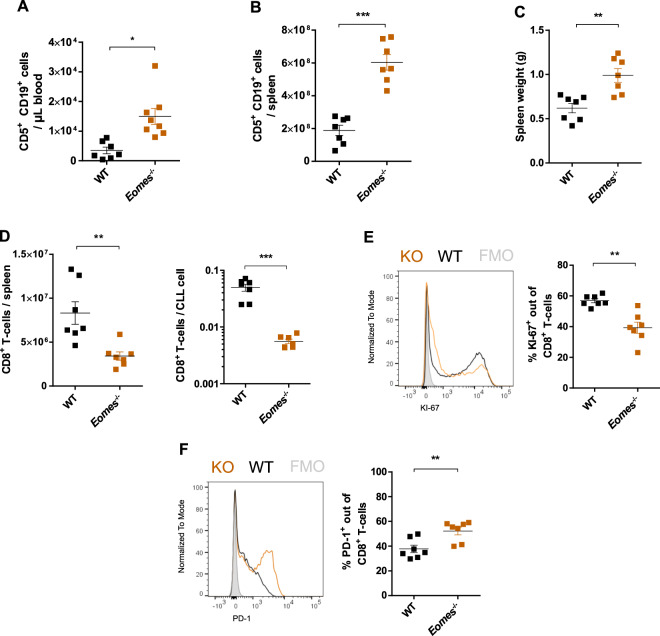
Lack of *Eomes* in the TME of TCL1 AT mice leads to impaired expansion of CD8^+^ T cells and enhanced CLL development. *Rag2*^−/−^ mice were irradiated and transplanted i.v. with 1 × 10^6^ bone marrow cells of wild-type (WT, *n* = 7) or *Eomes*^−/−^ (*n* = 7) origin on day -1, and after reconstitution of the hematopoietic system, with 1 × 10^7^ leukemic cells of Eµ-TCL1 mice. Blood and spleen samples of these mice were analyzed by flow cytometry after 4 weeks. **A** Absolute numbers of CD5^+^ CD19^+^ CLL cells in blood, and **B** in spleen. **C** Spleen weights of leukemic *Rag2*^−/−^ mice injected with WT or *Eomes*^−/−^ CD8^+^ T cells. **D** Absolute counts of CD8^+^ T cells (right) and relative counts per CLL cell (left) in spleen. **E** Representative histogram and percentages of KI67^+^ cells out of total CD8^+^ T cells in spleen. **F** Representative histogram and percentages of PD-1^+^ cells out of total CD8^+^ T cells in spleen. Graphs show mean with SEM, with each dot representing one mouse. Statistical analysis was performed using the Mann–Whitney’s test. *p value < 0.05, **p value < 0.01, ***p value < 0.001.

To analyze the role of *EOMES* in T-cell-mediated control of CLL, we generated *Eomes*^−/−^ BM chimeric mice, as previously described [20, 28]. Interestingly, mice with an *Eomes*-deficient hematopoietic system showed a higher tumor burden compared to *Eomes* WT chimeric mice at the experimental endpoint, 4 weeks after TCL1 AT. This was determined by an increased CD5^+^ CD19^+^ leukemic cell count in blood and spleen, as well as a higher spleen weight, compared to control mice (Fig. 3A–C). As this was associated with a lower total number of CD8^+^ T cells, both per spleen and per CLL cell, in *Eomes*^−/−^ BM chimera compared to controls (Fig. 3D), a diminished tumor control by CD8^+^ T cells seems a likely explanation for the enhanced tumor development. The decreased T-cell numbers in *Eomes*-deficient chimera were attributable to an impaired CD8^+^ T-cell expansion, as shown by a reduced percentage of KI67^+^ CD8^+^ T cells in *Eomes*^−/−^ mice (Fig. 3E). Analysis of the T-cell phenotype revealed a clear enrichment of effector T cells in both Eomes^−/−^ and control chimera, and no differences in the proportions of naïve, effector, and memory subsets (Supplementary Fig. 3A). Besides, no differences in the expression of the effector molecules granzyme B (GZMB), tumor necrosis factor alpha (TNFα), or interferon gamma (IFNγ) after ex vivo stimulation with PMA/ionomycin were observed (Supplementary Fig. 3B), suggesting that EOMES is not essential for effector cell differentiation. Intriguingly, the lack of EOMES was associated with a higher percentage of CD8^+^ T cells expressing PD-1 (Fig. 3F), but not LAG3 (Supplementary Fig. 3C), which might be secondary to more advanced disease associated with a higher antigenic load in the *Eomes*^−/−^ mice leading to more T-cell activation.

**Fig. 4:**
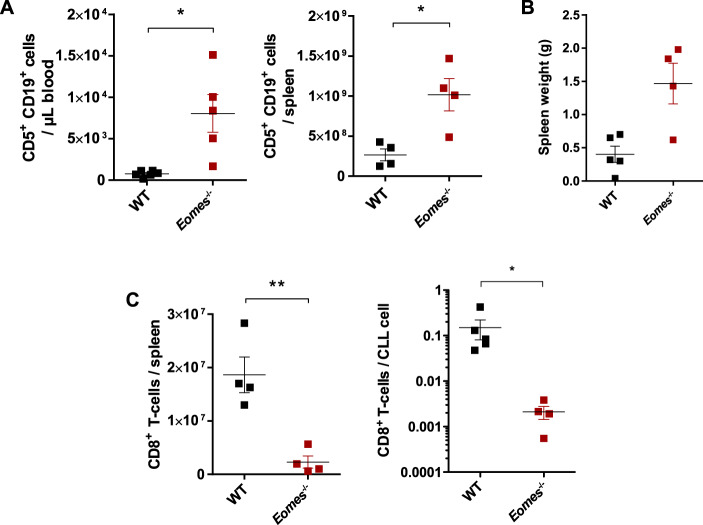
CD8^+^ T-cell-mediated CLL control in the TCL1 AT mouse model depends on EOMES. *Rag*2^−/−^ mice were transplanted i.v. with 1 × 10^6^ CD8^+^ T cells of wild-type (WT, *n* = 5) or *Eomes*^−/−^ origin (*n* = 5) on day -1. The following day, 1 × 10^7^ leukemic cells were injected i.p., and blood and spleen samples were analyzed by flow cytometry after 4 weeks. **A** Absolute numbers of CD5^+^ CD19^+^ CLL cells in blood (left) and in spleen (right). **B** Spleen weights and **C** absolute counts of CD8^+^ T cells as well as relative numbers of CD8^+^ T cells per CLL cell in spleen. Graphs show mean with SEM, with each dot representing one mouse. Statistical analysis was performed using the Mann–Whitney’s test. *p value < 0.05, **p value < 0.01.

To verify that these observed effects were intrinsic to CD8^+^ T cells and not induced by other *Eomes*-deficient cells in *Eomes*^−/−^ BM chimera, such as previously described for CD4^+^ T cells [28], we transferred *Eomes*^−/−^ or WT CD8^+^ T cells in *Rag2*^−/−^ mice and injected these mice 1 day later with Eµ-TCL1 leukemic cells. Similar to the results in the BM chimeric mice, leukemia development was significantly enhanced in mice that received *Eomes*-deficient CD8^+^ T cells compared to mice with WT T cells, with higher absolute numbers of CLL cells present in the blood, as well as an increased spleen weight and tumor content in this organ (Fig. 4A, B). In addition, *Eomes*-deficient CD8^+^ T cells expanded less in leukemic *Rag2*^−/−^ mice compared to WT CD8^+^ T cells, leading to significantly lower absolute T-cell counts and T-cell numbers per CLL cell in the spleen (Fig. 4C). As observed in BM chimeras, *Eomes*^−/−^ CD8^+^ T cells showed no change in the expression of GZMB, TNFα, or IFNγ after ex vivo stimulation with PMA/ionomycin (Supplementary Fig. 3D).

Overall, these observations suggest that EOMES is essential for CD8^+^ T-cell expansion and/or maintenance in mice that develop CLL-like disease. This impaired expansion is likely the reason for the limited CLL control in TCL1 AT mice.

## Discussion

Extensive research has highlighted the characteristics and pathological relevance of alterations in the T-cell compartment of the CLL microenvironment [18, 20, 28–31]. However, current immunotherapy approaches, including immune checkpoint blockade or CAR T-cell therapy, have very limited effects in CLL [32, 33]. Therefore, recent efforts focus on discerning and modulating the specific mechanisms responsible for the poor efficacy of and the resistance to these treatments in the CLL context, including T-cell exhaustion [18, 20, 34, 35].

In this study, we investigated the expression of EOMES transcription factor in tumor and T cells from PB and LN of CLL patients and its relation to T-cell exhaustion. Using the TCL1 AT mouse model, we further provide evidence for EOMES being essential for a T-cell-mediated tumor control.

Several SNPs located within the regulatory region of the *EOMES* gene and associated with increased risk for CLL (rs9880772) [1, 36] and Hodgkin’s Lymphoma (rs3806624) [37] have been identified through GWAS. Interestingly, the *EOMES* locus is a heterochromatic region in B cells [2]. Our analyses using histone marks and RNA-sequencing data confirmed that the *EOMES* locus is transcriptionally inactive in CLL and B cells across all investigated differentiation stages. Moreover, we showed that this region is solely euchromatic in T lymphocytes and NK cells. The functional characterization of a SNP located in a regulatory region of a gene and its role in CLL pathobiology is a major challenge and represents a limitation in our study as it remains unclear whether this SNP leads to differential expression of the gene. But as a functional role can be merely expected via T or NK cells, we hypothesize that the elevated risk for CLL in individuals with rs9880772 is linked to a reduced ability of these cells to control leukemia development.

Our results show that CD8^+^ T cells within the CLL TME express higher levels of EOMES in blood and especially in LNs of patients compared to HD. This increased EOMES expression is in line with the known skewed phenotype of CD8^+^ T cells toward activation-induced dysfunction in CLL, which suggests a role for EOMES in CD8^+^ T-cell-mediated control of CLL progression. Moreover, similar alterations could be observed in the Eμ-TCL1 transgenic as well as AT mouse models of CLL. Notably, Eμ-TCL1 mice, which heterogeneously develop a CLL-like disease over the course of 1 year, showed phenotypical changes in T_M_ cells, which might be indicative of an acquired memory to some tumor clones, similarly to chronic viral infections [38, 39]. Instead, more pronounced changes in the TCL1 AT model, especially in the effector compartment, might be due to a more acute immune reaction against the tumor. Crucially, our observation of EOMES levels being highest in exhausted CD8^+^ T cells is further supported by similar observations in other tumors [10–12, 40, 41].

A functional involvement of EOMES in driving T-cell exhaustion has been suggested by studies of subcutaneous tumor models [9]. However, in the CLL mouse model, we observed no major impact of EOMES on the differentiation or functional capacity of CD8^+^ T cells, which might be due to the overlapping function of EOMES with the phylogenetically related transcription factor T-BET [5, 8]. Our data, however, show that EOMES is required for efficient CD8^+^ T-cell expansion, which is in line with a previously described role of EOMES in enhancing CD8^+^ T-cell proliferation and survival and thereby, improving tumor control in a mouse model of cancer immunotherapy [42]. The importance of EOMES in adaptive immune control of tumors is further exemplified by a subset of CD4^+^ T cells that expresses EOMES and shows cytotoxic activity [43]. In line with this, we have recently identified an EOMES^+^ CD4^+^ T-cell subset, which is increased in CLL and co-expresses PD-1 and LAG3 [28]. Similarly, as observed for CD8^+^ T cells, EOMES deficiency in CD4^+^ T cells resulted in faster CLL progression in TCL1 AT mice. This data suggest that EOMES is important for both CD4^+^ and CD8^+^ T-cell-mediated control of CLL.

Altogether, these observations highlight the essential role that EOMES plays in T-cell expansion and proliferation. Accordingly, the investigation of EOMES target genes that impact on T-cell-mediated tumor control as means to improve immunotherapy outcomes is an important task for future research.

## Materials and methods

### Patient samples

Patient samples were obtained after informed consent of the patients and with the approval of study protocols by local ethics committees from the Department of Internal Medicine III of the University Clinic Ulm, the Department of Medicine V of the University Clinic Heidelberg, and Hospital Clínic of Barcelona according to the declaration of Helsinki. Age-matched healthy donor control blood samples were obtained from Biomex GmbH (Heidelberg, Germany) after informed consent. Clinical information of patients is provided in Supplementary Tables 1 and 2, and in ref. [20].

### Processing of patient samples

PB was collected in diamine tetraacetic acid (EDTA)-coated tubes (Sarstedt, Nümbrecht, Germany) and peripheral blood mononuclear cells were isolated by Ficoll (Biochrom, Berlin, Germany) density gradient centrifugation. Viable cells were frozen for long-term storage. For flow cytometry staining, samples were thawed and let rest for 3 h before further processing. LN samples were processed as previously described [44].

### Chromatin states

ChIP-seq data from six histone modifications (H3K4me3, H3K4me1, H3K27ac, H3K36me3, H3K27me3, and H3K9me3) of different healthy cell subtypes of the hematopoietic system were obtained from the Blueprint Epigenome Consortium (http://www.blueprint-epigenome.eu). The assignment of chromatin states was performed as previously described using the chromHMM pipeline [45, 46]. All profiles were corrected using their corresponding input. Chromatin states were assigned per 200-bp window.

### RNA-seq tracks

RNA-seq tracks from different hematopoietic cells were downloaded from the Blueprint Epigenome Consortium (http://www.blueprint-epigenome.eu). In particular, we used the bigWig tracks aligned to the GRCh38 reference genome using unique mappings and representing the expression of the negative strand (see http://dcc.blueprint-epigenome.eu/#/md/methods).

### Tumor models and adoptive T-cell transfer experiments

All mouse experiments were conducted according to institutional and governmental guidelines approved by the local authorities (Regierungspräsidium Karlsruhe, permit numbers: G25/16 and G98/16).

Eµ-TCL1 mice, (C. Croce, OH, USA [47]) bred on a pure C57BL/6N or J background, and *Rag2*^−/−^ mice were maintained under specific pathogen-free conditions at the central animal facility of the German Cancer Research Center (DKFZ). Analyzed mice were males and females of 47–49 weeks of age. The TCL1 AT mouse model has been previously described [28]. Briefly, 2–3 months old C57BL/6 female mice were i.v. injected with 1 × 10^7^ leukemic cells isolated from Eµ-TCL1 splenocytes. Tumor development was monitored in blood until endpoint.

For the generation of a T-cell-specific *Eomes* deletion, mice that carry loxP sites flanking the exons 2–5 of the *Eomes* gene [48] were crossed with a transgenic line expressing *Cre* under the *Lck* promoter [49]. *Lck-cre x Eomes*^*fl/fl*^ *x Foxp3-IRES-mRFP (FIR)* [6] x *Il10-GFP (tiger)* [50] (*Eomes*^−/−^ *knock-out* mice) or *Eomes*^*fl/fl*^ *x FIR x tige*r (WT) mice were used as source of Eomes KO or WT T cells, respectively. These mouse lines were maintained at the Max-Planck Institute of Immunobiology and Epigenetics (Freiburg, Germany).

T-cell adoptive experiments were performed as follows: 6–8-week-old female *Rag2*^−/−^ mice were i.v. injected with CD8^+^ T cells isolated from *Eomes*^−/−^ or *Eomes* WT mice using the EasySep^TM^ CD8^+^ T-cell isolation Kit (Stemcell Technologies, Vancouver, Canada) with a purity above 90%. Next day, mice were injected with 5 × 10^6^ T-cell depleted splenocytes from TCL1 AT mice. BM chimeras were generated in 6–8-week-old female *Rag2*^−/−^ mice, which lack T cells and B cells [51]. Mice were subjected to whole-body irradiation with two doses of 550 rads 3 h apart. Next day, mice were injected with 5 × 10^6^ T-cell-depleted BM cells from *Eomes*^−/−^ or *Eomes* WT mice. Following BM reconstitution after 6 weeks, mice were transplanted with malignant CLL cells of TCL1 AT mice, and leukemia development and the expansion and functional capacity of CD8^+^ T cells were investigated.

### Murine tissue sample preparation

PB was withdrawn via facial vein puncture and collected in EDTA-coated tubes and erythrocytes were lysed prior cell staining, as described below. Mice were euthanized with increasing concentrations of carbon dioxide (CO_2_). Collection and processing of spleen, BM, and inguinal LNs for generation of single-cell suspension have been described elsewhere [52].

### Flow cytometry analyses

In order to obtain absolute counts from whole blood, a defined volume of blood was stained for extracellular markers for 30 min at 4 °C. Next, lysis of erythrocytes was performed by addition of Red Blood Cell Lysis Buffer (BD Bioscience, Heidelberg, Germany) for 15 min at room temperature. Finally, cells were centrifuged, resuspended in PBS, and a defined amount of 123count eBeads^TM^ Counting Beads (ThermoFisher Scientific) was added before sample measurement.

Single-cell suspensions were stained with cell-surface antibodies and fixable viability dye for 30 min at 4 °C. Cells were subsequently washed twice with FACS buffer. For staining of intranuclear proteins, surface-stained cells were fixed with eBioscience^TM^ Foxp3/Transcription Factor Staining Buffer Set (ThermoFisher Scientific) for 30 min at room temperature. After additional washing steps, cells were permeabilized using eBioscience^TM^ Permeabilization Buffer (ThermoFisher Scientific) and stained for 30 min at room temperature. Samples were kept at 4 °C in dark conditions until acquisition.

For intracellular cytokine staining, single-cell suspensions were cultured ex vivo with PMA and Ionomycin and protein transport inhibitor (Cell Stimulation Cocktail and Inhibitor Cocktail respectively, both from eBioscience^TM^, ThermoFisher Scientific) for 6 h at 37 °C and 5% CO_2_. Next, cells were washed and stained as described above.

The complete list of antibodies used in this study is provided as Supplementary Table 3.

Samples were measured using BD FACS Canto, BD LSR II, or BD LSR Fortessa machines, and data were analyzed with FlowJo X 10.0.7 software (Flowjo, Ashland, OR, USA). Naïve, memory, and effector subsets were defined as CD127^+^ CD44^−^, CD127^+^ CD44^+^, and CD127^−^CD44^+^, respectively. Cell clustering was performed using t-SNE algorithm from FlowJo, using pre-gated concatenated CD8^+^ T cells from four TCL1 AT mice. Markers CD127, CD44, EOMES, PD-1, LAG3, TIGIT, and CD244 were considered for clustering.

### Statistical analyses

Sample size was determined based on expected variance of read-out. No samples or animals were excluded from the analyses. No randomization or blinding was used in animal studies. The statistical test used for each data set is indicated in the figure legends. The Mann–Whitney test was used to investigate the significance of the differences between sample groups. One-way analysis of variance was used to determine differences between more than two groups. *P* values of <0.05 were considered statistically significant. All graphs show means ± standad error of the mean (SEM), unless otherwise indicated.

## Supplementary information

Supplementary Figure Legends and Tables

Supplementary Figure 1

Supplementary Figure 2

Supplementary Figure 3
