# Supplementary material for: EOMES is essential for antitumor activity of CD8+ T cells in chronic lymphocytic leukemia
Source: Leukemia. 2021 Mar 17;35(11):3152–62. doi: 10.1038/s41375-021-01198-1 (PMC8550953; doi:10.1038/s41375-021-01198-1)
Supplement: Supplementary file 1 — Supplementary Figure Legends and Tables [file 41375_2021_1198_MOESM1_ESM.docx]

**Legends to Supplementary Figures**

**Supplementary Figure 1: EOMES expression in CLL cells and CD8^+^ T-cells.** EOMES expression was analyzed by flow cytometry in CD8^+^ T-cells from blood samples of CLL patients (n = 12) and healthy donors (HD, n = 8), as well as from CLL and reactive lymph node samples. **A)** Representative gating strategy to define CLL and T-cells in CLL lymph nodes (LN) and peripheral blood (PB) by flow cytometry. CD8^+^ T-cells are further gated as T_EF_ (CCR7^-^ CD45RO^-^), T_EM_ (CCR7^-^ CD45RO^+^), T_N_ (CCR7^+^ CD45RO^-^), and T_CM_ (CCR7^+^ CD45RO^+^) cells. CLL cells were defined as CD3^-^ HLA-DR^+^ cells. **B)** Representative histogram of EOMES expression in CD8^+^ T-cells and CLL cells from PB of one CLL patient. **C-D)** Percentage of EOMES^+^ cells out of **C)** total as well as **D)** T_EM_, T_CM_ and T_N_ CD8^+^ T-cells from PB of CLL patients and healthy donors (HD). **E)** Normalized mean fluorescence intensity (nMFI) of EOMES in PD-1^+^ and PD-1^-^ CD8^+^ T_EM_ cells from CLL and HD PB. **F)** Percentage of EOMES^+^ PD-1^+^ cells out T_EM_ CD8^+^ T-cells in CLL and HD PB, as well as **G)** CLL LN (n = 6) versus reactive lymph node (rLN, n = 9) samples. **H)** MFI of EOMES in T_EM_ and T_EF_ CD8^+^ T-cells from rLN and CLL LN samples. FMO = Fluorescence Minus One. Graphs display mean ± SEM, with each dot representing one patient or donor. Mann-Whitney test was used for statistical analysis. * p-value <0.05, ** p-value < 0.01.

**Supplementary Figure 2: CD8^+^ T-cells in Eµ-TCL1 and TCL1 AT mice. A)** Representative gating strategy to define T-cells from splenocytes in the Eµ-TCL1 and TCL1 AT mouse models. **B)** Percentages of naïve, memory and effector subsets out of total CD8^+^ T-cells in Eµ-TCL1 mice (green) compared to age- and sex-matched wild-type (WT) littermates (black). **C)** Pearson correlation plot of absolute counts (AC) of CD8^+^ T-cells and CLL cells in spleen of Eµ-TCL1 mice. **D)** Representative histogram and normalized MFI values of EOMES in naïve (CD127^+^ CD44^-^), memory (CD127^+^ CD44^+^), effector (CD127^-^ CD44^+^), and PD1^+^ LAG3^+^ CD8^+^ T-cell subsets in the spleen of primary Eµ-TCL1 leukemic mice (n = 8). **E)** Normalized MFI values of EOMES in effector and memory PD-1^-^ LAG3^-^ versus PD-1^+^ LAG3^+^ CD8^+^ T-cells (n = 48). **F)** Percentages of naïve, memory and effector subsets out of total CD8^+^ T-cells in TCL1 AT mice (turquoise) compared to age- and sex-matched WT littermates (black). **G)** Representative histogram and normalized MFI values of EOMES in naïve (CD127^+^ CD44^-^), memory (CD127^+^ CD44^+^), effector (CD127^-^ CD44^+^), and PD1^+^ LAG3^+^ CD8^+^ T-cell subsets in the spleen of end-stage TCL1 AT mice. **H)** Normalized MFI values of EOMES in effector and memory PD-1^-^ LAG3^-^ versus PD-1^+^ LAG3^+^ CD8^+^ T-cells in the spleen of end-stage TCL1 AT mice. Graphs display mean ± SEM, with each dot representing one mouse. Mann-Whitney test was used for statistical analysis. * p-value < 0.05, ** p-value < 0.01, *** p-value < 0.001, **** p-value < 0.0001.

**Supplementary Figure 3: Lack of *Eomes* does not alter CD8^+^ T-cell subset distribution or functional capacity. A, B)** Rag2^-/-^ mice were irradiated and injected with bone marrow cells from wild-type (WT) or *Eomes*^-/-^ mice on day -1, and after reconstitution of the hematopoietic system, with TCL1 leukemic cells. Splenocytes of these mice were analyzed by flow cytometry after 4 weeks. **A)** Percentages of naïve, memory and effector subsets out of total CD8^+^ T-cells. **B)** Percentages of GZMB^+^, IFNγ^+^ and TNFα^+^ cells out of total CD8^+^ T-cells after *ex vivo* stimulation with PMA and ionomycin for 6 hours. **C)** Percentage of LAG3^+^ among CD8^+^ T-cells. **D)** *Rag2*^-/-^ mice were injected with WT or *Eomes*^-/-^ CD8^+^ T-cells on day -1 and with TCL1 leukemic cells on day 0. After 4 weeks, percentages of GZMB^+^, IFNγ^+^ and TNFα^+^ cells out of total splenic CD8^+^ T-cells after *ex vivo* stimulation with PMA and ionomycin for 6 hours were analyzed by flow cytometry. Graphs display mean ± SEM, with each dot representing one mouse. Mann-Whitney test was used for statistical analysis.

**Supplementary Table 1: Clinical information for blood samples of CLL patients and healthy controls (HC)**

|  | HC | CLL |
| --- | --- | --- |
| Number of samples | 8 | 12 |
| Sex | 50.0 % female (4/8) | 58.3 % female (7/12) |
| Age (years) | mean: 60.6 | mean: 57.3 |
|  | median: 62 | median: 54 |
| CMV IgG positivity | 80 % CMV IgG^+^ (4/5) | 45.5 % CMV IgG^+^ (5/11) |
| Binet stage |  | 75.0 % A (9/12) |
|  |  | 16.7 % B (2/12) |
|  |  | 8.3 % C (1/12) |
| IGHV state |  | 66.7 % mutated (8/12) |
| Chromosomal aberration |  | 58.3 % del13q14.3 (8/12) |
|  |  | 25.0 % normal karyotype (3/12) |
|  |  | 8.3 % IGH translocation (1/12) |
| TP53 mutation |  | 10.0 % (1/10) |
| Prior treatment |  | 0.0 % (0/12) |
| rs988072 phenotype |  | GG (2/12) GA (6/12) AA (1/12)  NA (3/12) |

Note: GG/ GA (non-risk allele), AA (risk allele); NA = not available; CMV = cytomegalovirus

**Supplementary Table 2: Information for lymph node samples of CLL patients and reactive lymph nodes (RLN) of healthy controls**

|  | CLL | RLN |
| --- | --- | --- |
| Number of samples | 7 | 9 |
| Clinical situation | Initial diagnosis: 6/7 | NA |
|  | Relapse: 1/7 |  |
| Age (years) | Mean: 72 | Mean: 39 |
|  | Median: 74 | Median: 38 |
| sex | 0 % female (0/7) | 22.2 % female (2/9) |
| rs988072 phenotype | GG (2/7) GA (3/7) AA (1/7)  NA (1/7) |  |

Note: GG/ GA (non-risk allele), AA (risk allele); NA = not available

**Supplementary Table 3: List of flow cytometry antibodies**

| Marker | Fluorocrome | Species reactivty | Clone | Supplier | Reference |
| --- | --- | --- | --- | --- | --- |
| CCR7 | BV605 | Human | 3D12 | BD Bioscience | 563711 |
| CCR7 | V450 | Human | 150503 | BD Bioscience | 560863 |
| CD107A | PE | Mouse | 1D4B | eBioscience | 12-1071-83 |
| CD11B | PerCpcy5.5 | Mouse | M1/70 | eBioscience | 45-0112-82 |
| CD127 | PE | Mouse | [A7R34](https://www.biolegend.com/en-us/search-results?Clone=A7R34) | Biozol Diagnostica | BLD-135010 |
| CD127 | PE-Dazzle | Mouse | [A7R34](https://www.biolegend.com/en-us/search-results?Clone=A7R34) | Biozol Diagnostica | BLD-135032 |
| CD127 | BV605 | Mouse | [A7R34](https://www.biolegend.com/en-us/search-results?Clone=A7R34) | Biozol Diagnostica | BLD-135041 |
| CD127 | PE-Cy7 | Mouse | A7R34 | Biolegend | 25-1371-82 |
| CD19 | PE | Mouse | 1D3 | eBioscience | 12-0193-83 |
| CD19 | PE-Dazzle | Mouse | 6D5 | Biolegend | 115554 |
| CD19 | FITC | Mouse | 1D3 | eBioscience | 11-0193-86 |
| CD3 | V450 | Mouse | 500A2 | BD Bioscience | 560801 |
| CD3 | PerCP/Cyanine5.5 | Human | OKT3 | Biolegend | 317336 |
| CD3E | BV605 | Mouse | 145-2C11 | Biolegend | BLD-100351 |
| CD3E | FITC | Mouse | 145-2C11 | Biolegend | 100306 |
| CD4 | PerCP-Cy5.5 | Mouse | RM4-4 | Biolegend | 116012 |
| CD4 | APC | Mouse | RM4-4 | Biozol Diagnostica | BLD-100516 |
| CD4 | APC-Cy7 | Mouse | RM4-5 | Biolegend | 100526 |
| CD4 | BUV395 | Human | SK3 | BD Bioscience | 563552 |
| CD44 | FITC | Human/ Mouse | IM7 | eBioscience | 11-0441-85 |
| CD44 | AF700 | Mouse | IM7 | eBioscience | 56-0441-82 |
| CD45 | AF700 | Mouse | 30-F11 | Biolegend | 103128 |
| CD45 | BV711 | Mouse | 30-F11 | Biolegend | 103147 |
| CD45RO | APC-Cy7 | Human | UCHL1 | Biolegend | 304227 |
| CD5 | APC | Mouse | 53-7.3 | Biolegend | 100626 |
| CD5 | BV605 | Mouse | 53-7.3 | BD Bioscience | 563194 |
| CD5 | PE-Cy7 | Human | L17F12 | BD Bioscience | 348790 |
| CD8 | FITC | Human | HIT8a | Biolegend | 300905 |
| CD8 | BV605 | Human | RPA-T8 | Biolegend | 301039 |
| CD8A | APC-Cy7 | Mouse | 53-6.7 | Biolegend | 100714 |
| CD8A | BV605 | Mouse | 53-6.7 | Biolegend | 100744 |
| CX3CR1 | PE-Dazzle | Mouse | SA011F11 | Biolegend | 149014 |
| EOMES | PerCP- efluor710 | Mouse | Dan11mag | eBioscience | 46-4875-80 |
| EOMES | eFluor®660 | Mouse | WD1928 | eBioscience | 50-4877-42 |
| EOMES | PE | Human | WD1928 | eBioscience | 12-4877-42 |
| EOMES | eFluor 660 | Human | WD1928 | BD Bioscience | [50-112-8864](https://www.fishersci.com/shop/products/eomes-mouse-anti-human-efluor-660-clone-wd1928-ebioscience-2/501128864) |
| GZMB | eFluor®660 | Mouse | NGZB | eBioscience | 50-8898-82 |
| HLA-DR | BV 711 | Human | L243 | Biolegend | 307643 |
| IFNG | PerCP-Cy5.5 | Mouse | XMG1.2 | eBioscience | 45-7311-82 |
| KI-67 | FITC | Mouse | SolA15 | eBioscience | 11-5698-82 |
| LAG3 | PE | Mouse | eBioC9B7W | eBioscience | 12-2231-82 |
| PD-1 | PE-Cy7 | Mouse | RPM1-30 | Biolegend | 109110 |
| PD-1 | PE-Dazzle | Mouse | RPM1-30 | Biolegend | 109116 |
| PD-1 | BV-421 | Human | EH12.2H7 | Biolegend | 329919 |
| PD-1 | PE-Cy7 | Human | EH12.2H7 | Biolegend | 329918 |
| TNF | eflour405 | Mouse | MP6-XT22 | eBioscience | 48-7321-82 |
