# Supplementary figures and images for: EOMES is essential for antitumor activity of CD8+ T cells in chronic lymphocytic leukemia
Source: Leukemia. 2021 Mar 17;35(11):3152–62. doi: 10.1038/s41375-021-01198-1 (PMC8550953; doi:10.1038/s41375-021-01198-1)

# Supplementary Figure 1

**A**

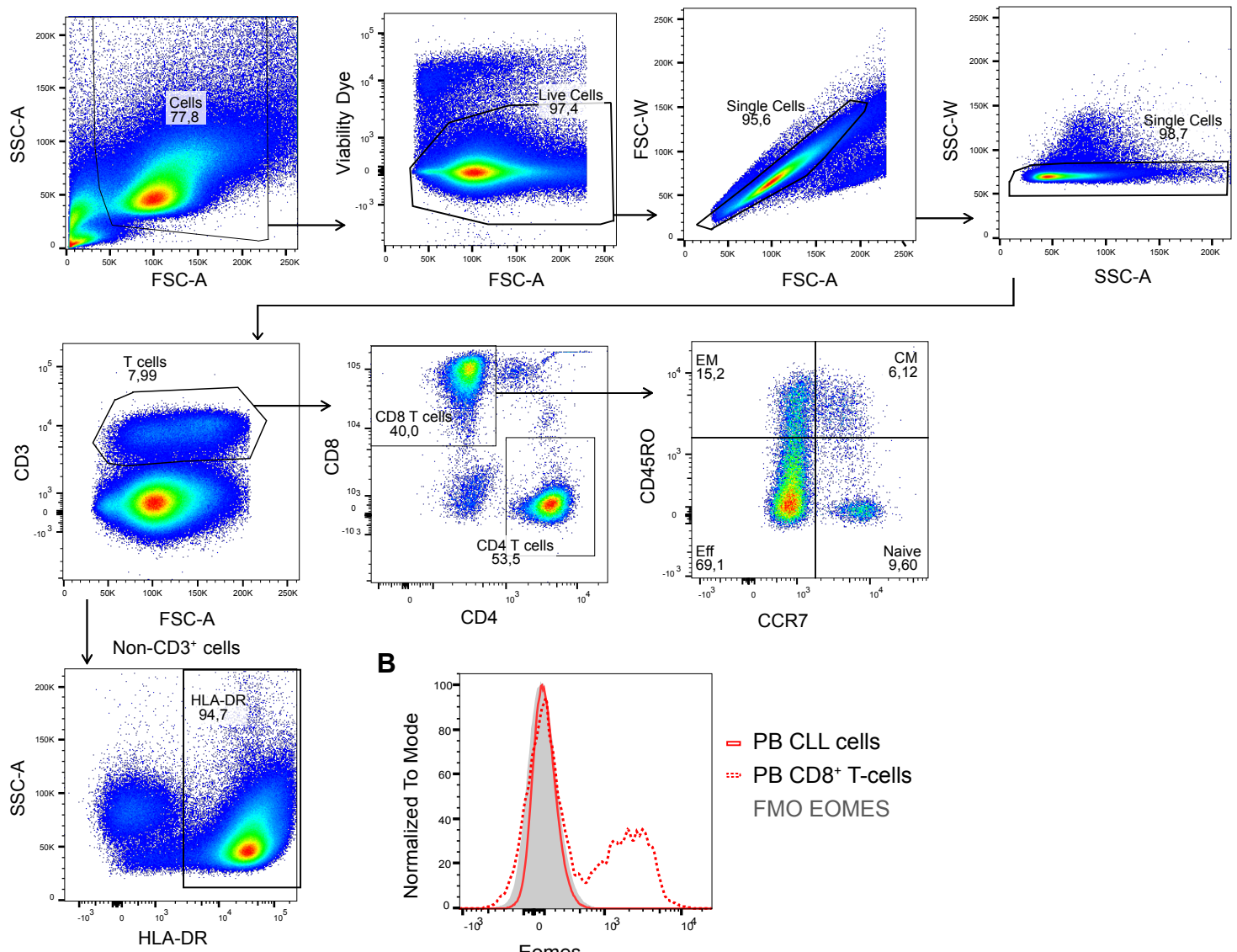

**B**

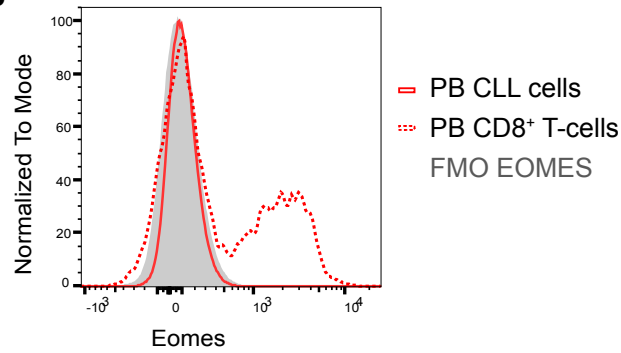

**C**

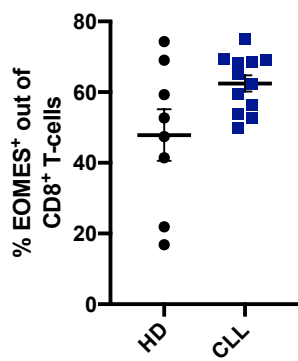

**D**

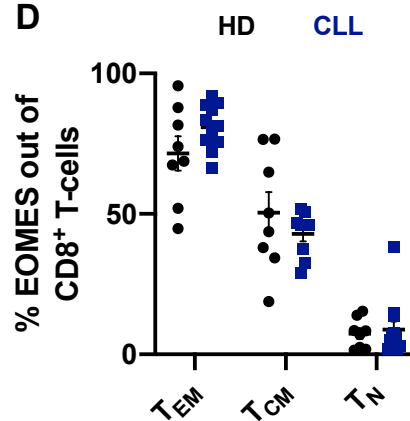

**E**

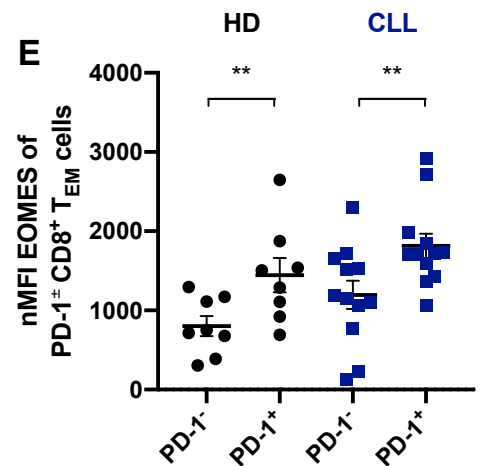

**F**

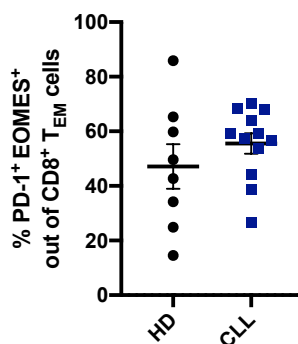

**G**

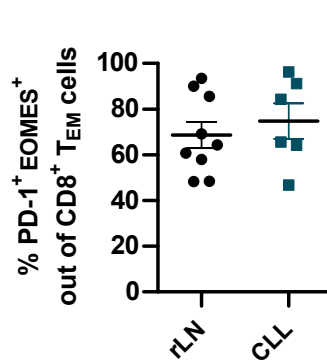

**H**

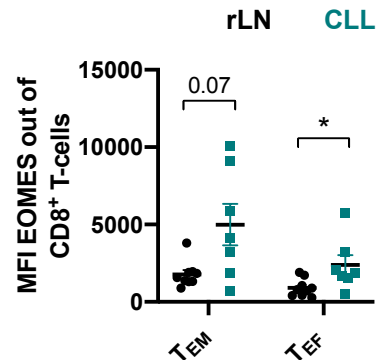

Supplement: Supplementary file 2 — Supplementary Figure 1 [file 41375_2021_1198_MOESM2_ESM.pdf]

**Supplementary Figure 2**

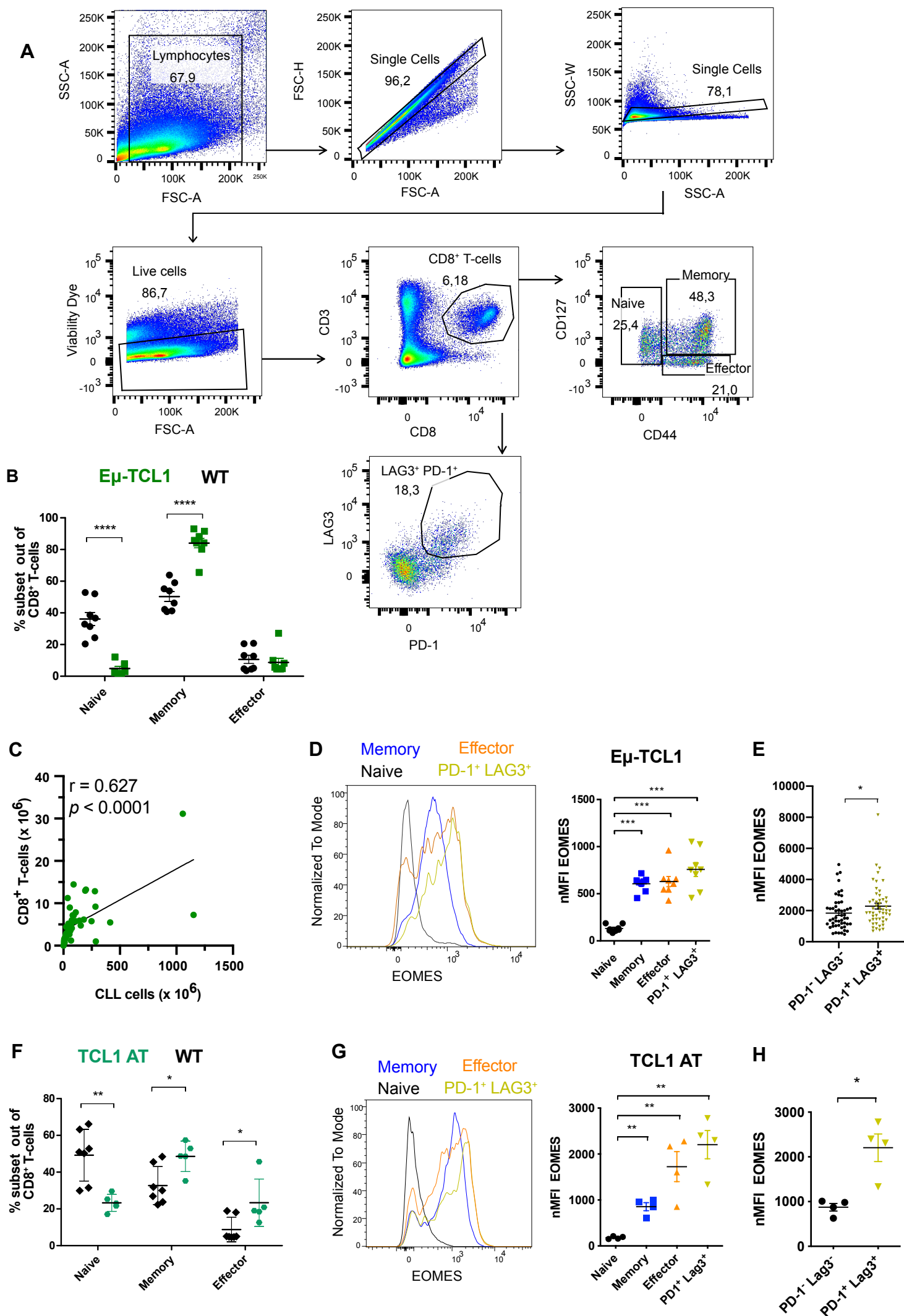

Supplement: Supplementary file 3 — Supplementary Figure 2 [file 41375_2021_1198_MOESM3_ESM.pdf]

Supplementary Figure 3

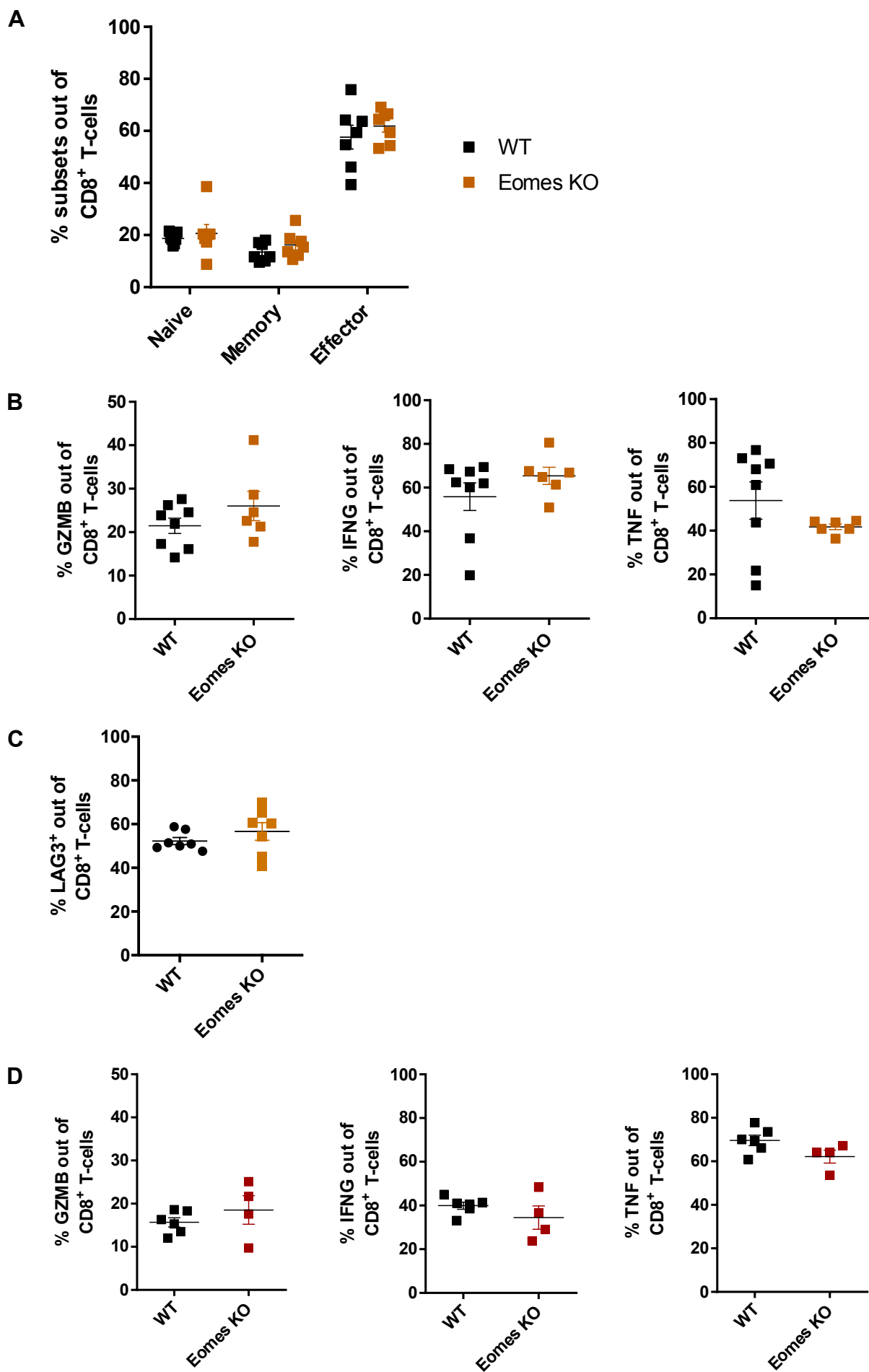

Supplement: Supplementary file 4 — Supplementary Figure 3 [file 41375_2021_1198_MOESM4_ESM.pdf]
